# hERG-deficient human embryonic stem cell-derived cardiomyocytes for modelling QT prolongation

**DOI:** 10.1186/s13287-021-02346-1

**Published:** 2021-05-07

**Authors:** Yun Chang, Ya-nan Li, Rui Bai, Fujian Wu, Shuhong Ma, Amina Saleem, Siyao Zhang, Youxu Jiang, Tao Dong, Tianwei Guo, Chengwen Hang, Wen-jing Lu, Hongfeng Jiang, Feng Lan

**Affiliations:** 1 Beijing Laboratory for Cardiovascular Precision Medicine, The Key Laboratory of Biomedical Engineering for Cardiovascular Disease Research, The Key Laboratory of Remodeling-Related Cardiovascular Disease, Ministry of Education, Beijing Anzhen Hospital, Capital Medical University, Research Institute Building, Room 323, 2 Anzhen Road, Chaoyang District, Beijing, 100029 China; 2 Beijing Institute of Heart, Lung and Blood Vessel Diseases, Beijing, 100029 China; 3 Department of Cardiology, Peking University Third Hospital, Beijing, 100191 China; 4 State Key Laboratory of Cardiovascular Disease, National Center for Cardiovascular Diseases, Fuwai Hospital, Key Laboratory of Application of Pluripotent Stem Cells in Heart Regeneration, Chinese Academy of Medical Sciences and Peking Union Medical College, Beijing, China

**Keywords:** Human ether-a-go-go-related gene, *KCNH2*, hESCs, QT prolongation, CRISPR/Cas9

## Abstract

**Background:**

Long-QT syndrome type 2 (LQT2) is a common malignant hereditary arrhythmia. Due to the lack of suitable animal and human models, the pathogenesis of LQT2 caused by *human ether-a-go-go-related gene* (hERG) deficiency is still unclear. In this study, we generated an hERG-deficient human cardiomyocyte (CM) model that simulates ‘human homozygous hERG mutations’ to explore the underlying impact of hERG dysfunction and the genotype–phenotype relationship of hERG deficiency.

**Methods:**

The *KCNH2* was knocked out in the human embryonic stem cell (hESC) H9 line using the CRISPR/Cas9 system. Using a chemically defined differentiation protocol, we obtained and verified hERG-deficient CMs. Subsequently, high-throughput microelectrode array (MEA) assays and drug interventions were performed to characterise the electrophysiological signatures of hERG-deficient cell lines.

**Results:**

Our results showed that *KCNH2* knockout did not affect the pluripotency or differentiation efficiency of H9 cells. Using high-throughput MEA assays, we found that the electric field potential duration and action potential duration of hERG-deficient CMs were significantly longer than those of normal CMs. The hERG-deficient lines also exhibited irregular rhythm and some early afterdepolarisations. Moreover, we used the hERG-deficient human CM model to evaluate the potency of agents (nifedipine and magnesium chloride) that may ameliorate the phenotype.

**Conclusions:**

We established an hERG-deficient human CM model that exhibited QT prolongation, irregular rhythm and sensitivity to other ion channel blockers. This model serves as an important tool that can aid in understanding the fundamental impact of hERG dysfunction, elucidate the genotype–phenotype relationship of hERG deficiency and facilitate drug development.

**Supplementary Information:**

The online version contains supplementary material available at 10.1186/s13287-021-02346-1.

## Background

The *KCNH2* encodes the voltage-gated K^+^ channel α subunit, Kv11.1, which is known as the *human ether-a-go-go-related gene* (hERG) [1]. The hERG channel constitutes a rapidly activated delayed rectifier K^+^ channel (IKr) in the heart [2] and is responsible for myocardial cell repolarisation when the cardiac action potential ends [3]. Previous evidence shows that *KCNH2* mutations are associated with hereditary long-QT syndrome type 2 (LQT2) [4]. The prevalence of LQT2 accounts for 25–40% of all long-QT syndrome (LQTS) types and is one of the most common inherited arrhythmias [5]. Generally, LQT2 has the characteristics of a prolonged QT interval and action potential duration (APD), which predispose patients to torsade de pointes, a type of polymorphic ventricular tachycardia and sudden cardiac death [6–8].

In most studies, to better understand the pathogenesis, the establishment of models that reveal disease mechanisms is crucial. Surprisingly, to date, although human homozygous hERG mutations have been reported, an appropriate model of human hERG deficiency has not been established. Transgenic mice expressing an hERG dominant-negative construct exhibited mild APD prolongation in individual myocytes, but QT prolongation was not observed in intact animals [9]. Targeted disruption of Erg1a in mice led to a homozygous embryonic lethal phenotype, whereas heterozygotes showed slight QT prolongation [10]. Since previous models failed to reproduce the disease phenotype, we should consider that the human heart is distinctly different from the murine heart. Mice have a faster heart rate (500–700 bpm) than humans, which represents different action potentials and repolarising K^+^ currents. In mice, the dominant cardiac repolarisation K^+^ currents are fast and slow transient outward currents and delayed rectifier voltage-gated K^+^ currents (IK, slow1 and IK, slow2), while the role of IKr is minimal [11–13]. Thus, the need to establish an hERG-deficient model to determine the pathological mechanism of this disease is urgent.

Recently, cardiomyocytes (CMs) derived from human embryonic stem cells (hESCs)/human pluripotent stem cells (hPSCs) have been used for modelling various hereditary cardiomyopathies [14, 15]. Numerous studies have shown that hESC and hPSC have high similarity [16–18]. LQTS was one of the first cardiac diseases that was recapitulated in hPSC models [19]. To date, cell models derived from LQTS mutation carriers have been widely used in mechanistic studies and for drug screening [20–22]. However, cell lines from different individuals contain additional genetic variants, which may limit the observation of genotype–phenotype relationships. Furthermore, there are several hERG heterozygous mutations, diverse phenotype of loss-of-function and different mutation mechanisms [23–25], which makes the research on the pathological mechanisms of hERG functional dysfunction more complicated. Furthermore, in individuals with heterozygous mutations, the presence of wild-type (WT) alleles may affect repolarisation. In cases that were heterozygous for R176W in 293 T cells, hERG tail current density was reduced by ~ 75% [26]. Nevertheless, in hPSC-CMs from a heterozygous R176W individual, a ~ 43% decrease in IKr density was observed [27]. The reasons for this observed discrepancy in cases with the same mutation are unknown but may involve the differential expression of WT and mutant alleles. Therefore, an appropriate hERG-deficient model, rather than mutation-based models, is desperately needed to determine the well-defined genotype–phenotype correspondence.

Here, we generated an hERG-deficient human CM model using the CRISPR/Cas9 system. After differentiation into CMs and characterisation by electrophysiology and drug intervention, the cell lines exhibited QT prolongation, irregular rhythm, early afterdepolarisation (EAD) occurrence and IKr current insensitivity. Additionally, nifedipine and magnesium chloride (MgCl_2_) could reverse the observed phenotype. Therefore, this model is appropriate for elucidating the pathogenesis of hERG dysfunction, defining genotype–phenotype correspondence and facilitating drug development.

## Methods

### Cell culture and cardiac differentiation

The human embryonic stem cell-H9 line (hESC-H9; provided by WiCell Institute Inc., Madison, WI, USA)/human pluripotent stem cell (hPSC, provided by Cellapy: CA1002008, Beijing, China) and the derived cells were cultured primarily in E8 medium (Cellapy, China). The cells were passaged with 0.5-mM EDTA–PBS solution (Cellapy) when the cells reached 80% confluence. CM differentiation was carried out using small molecule-based methods [28]. When spontaneous beating was obvious, hESC-CMs were purified by the lactic acid metabolism selection method [29].

### Genome editing

*KCNH*2 single-stranded guide RNA (sgRNA) (GCATCGACATGAACGCG) was designed using an online tool (http://crispr.mit.edu/). We electroporated the epiCRISPR vector and sgRNA (100-μl electrotransformation solution (Cellapy) plus 2.5-μg *KCNH2* gRNA plasmid) into the cells using the 4D nuclear receptor system and the CA137 programme (Lonza, Germany). The transfected cells were seeded in 6-well plates and cultured overnight in E8 medium containing 10 μM of Rho kinase inhibitor Y-27632. The medium was changed the next day. Drug (puromycin) selection was initiated after 72 h of transfection at a lower concentration of 0.1 μg/ml for the first hour and then at 0.3 μg/ml until the transfected lines were stable. The surviving cells were collected in 48-well plates and amplified for polymerase chain reaction (PCR) screening.

### Western blot

Cells were rinsed in PBS (Corning, USA) and harvested in a CardioEasy CM dissociation buffer (Cellapy). The cells were then rinsed again in PBS and pelleted by centrifugation twice at 1200 rpm for 5 min each time. After removing the supernatant, SDS-PAGE protein-loading buffer (Beyotime, China) was added, and the cells were lysed by sonication and heat-denatured. According to the molecular weight of the protein, we configured a 10% separation gel and a 4% concentration gel for electrophoresis and performed gel transfer to a polyvinylidene difluoride membrane using a gel transfer device (Bio-Rad) over 120 min. Then, we blocked the membrane with 5% skimmed milk for 1 h at room temperature. The membrane was incubated with the primary antibody overnight at 4 °C, followed by incubation with the secondary antibody for 2 h at room temperature.

### Immunofluorescence

Cells were seeded in 24-well plates, fixed with 4% paraformaldehyde (PFA) (Solarbio, China) for 10 min, permeabilised with 0.5% Triton X-100 (Sigma, USA) for 15 min and blocked with 3% BSA (Sigma) at room temperature for 30 min. The cells were incubated with the prepared primary antibody overnight at 4 °C. The following day, the cells were rinsed three times in 1× PBS for 5 min each time. The cells were incubated with the secondary antibody at room temperature in the dark for 2 h and then rinsed three times in 1× PBS for 5 min each time. Data were collected using a confocal microscope (Leica DMI 4000B).

### RNA extraction and quantitative reverse transcription PCR (qRT-PCR)

Total cellular RNA was extracted with TRIzol (Invitrogen, USA) and treated with DNase I (Beyotime, China) for approximately 30 min at 37 °C to eliminate DNA contamination. RNA was reverse transcribed using the Prime-Script^TM^ reverse transcription system (TaKaRa, Japan). Relative gene expression levels were examined by qRT-PCR using the iCycler iQ5 (Bio-Rad, USA) with TB Green™Premix Ex Taq™II (Takara). The relative quantification was calculated according to the ▵CT method. All primer sequences are listed in Table S1.

### Flow cytometry

CMs were dissociated into a single cell suspension in a CardioEasy CM dissociation buffer. Cells were then rinsed in PBS, fixed in 4% PFA for 10 min and permeabilised in 0.5% Triton X-100 for 15 min. The primary antibody was applied for 60 min, followed by incubation with the secondary antibody for 30 min at room temperature. The samples were then rinsed in PBS and assessed using FACS analysis (EPICS XL, Beckman).

### Microelectrode array (MEA) analysis

CMs were digested in a CardioEasy CM dissociation buffer, after which 2 × 10^4^ cells were plated on a microelectrode array (MEA) pre-coated with 5% Matrigel (Cellapy). The next day, 300-μl medium was added to each well. After the CMs resumed spontaneous beating, the experimental data were recorded on a Maestro EDGE (Axion Biosystems, Inc., Atlanta, USA) according to the MEA manual. Cardiac Analysis Tool, AxIS Navigator, AxIS data export tool and Origin were used to analyse the data.

### Data analysis and statistics

All experiments were repeated at least 3 times with duplicate samples. All data are displayed as the mean ± standard error of mean. Two-sided Student’s *t* test and one-way ANOVA were used to determine statistical significance, and *P* < 0.05 was considered statistically significant.

## Results

### Establishment of homozygous hERG-deficient hESCs

**Fig. 1:**
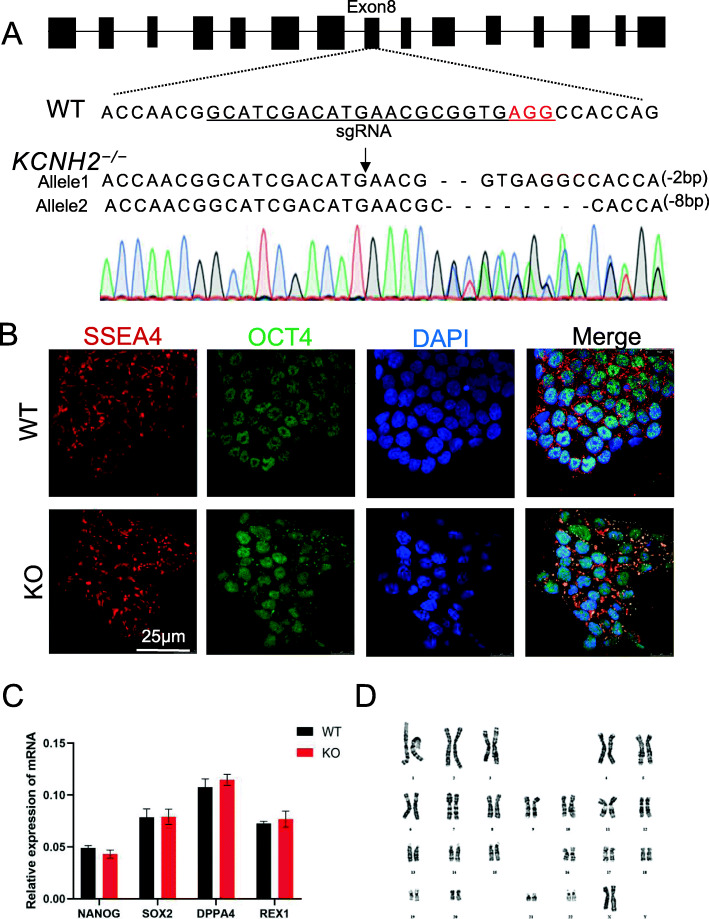
hERG deficiency did not affect the pluripotency of hESCs. **a** Pattern diagram of *KCNH2* knockout demonstrating the genic positions of their editing sites. **b** Pluripotency markers *SSEA4* and *OCT4* of cell lines immunofluorescent staining. Scale bar = 25 μm. **c** qRT-PCR analysis of pluripotency-related genes in WT and *KCNH2*^−/−^. **d** Karyotype analysis revealed a normal karyotype of 46 chromosomes in *KCNH2*^−/−^

We established an hERG-deficient cell model from the hESC-H9 cell line using the CRISPR/Cas9 system [30]. First, we designed a highly specific sgRNA to target the *KCNH2*. Next, hESC-H9 cells were subjected to electroporation with a plasmid containing sgRNA and Cas9, followed by puromycin screening. To assess the editing features of cell colonies, we analysed colonies using PCR and Sanger sequencing. Finally, we selected homozygous (biallelic mutations) colonies with − 2 bp and − 8 bp (Fig. 1a). In addition, to determine whether *KCNH2* knockout played the same role in different stem cell lines, we also established a *KCNH2*^−/−^hPSC cell line using the same method (data not shown). The *KCNH2*^−/−^ colonies exhibited normal morphology (Fig. S1A). In addition, we found that *KCNH2*^−/−^ lines expressed the pluripotency markers *OCT4* and *SSEA4* (Fig. 1b). Similarly, gene expression analysis confirmed the expression of pluripotency genes (*NANOG, SOX2, DPPA4* and *REX1*) (Fig. 1c). *KCNH2*^−/−^ lines also had a normal karyotype (Fig. 1d). A teratoma formation assay revealed that *KCNH2*^−/−^ cell lines exhibited stem cell properties (Fig. S1A).

### hERG-deficient hESCs can differentiate into CMs

**Fig. 2:**
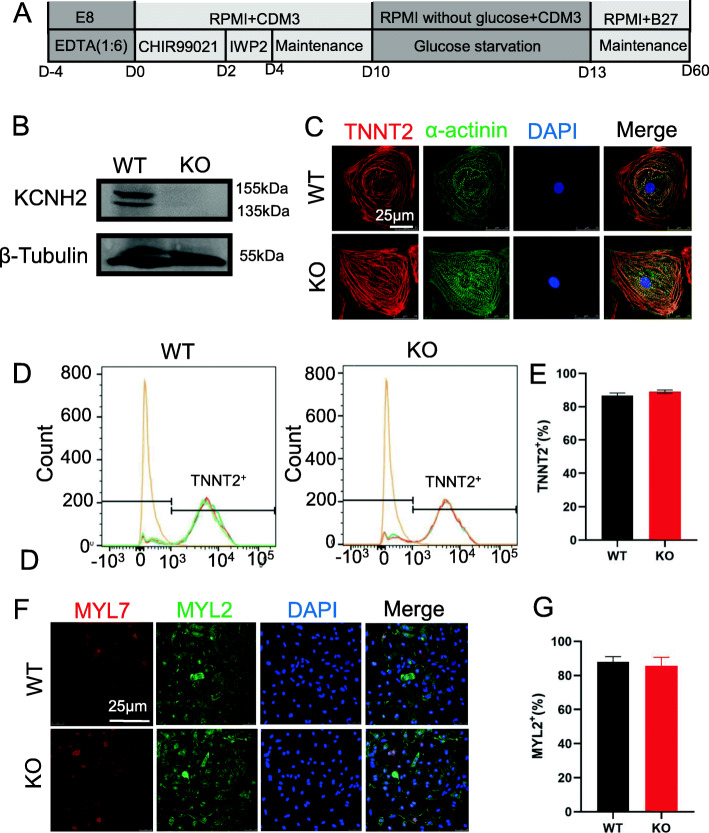
hERG deficiency did not affect the differentiation of hESC-CMs. **a** Schematic illustration of hESCs in vitro differentiation protocols using small molecule-based methods. **b** The hERG expression assessed by western blotting analysis. **c** Immunofluorescence staining of cardiomyogenic differentiation markers *TNNT2* and α-actinin. Scale bar = 25 μm. **d**, **e** Flow cytometry analyses of CMs marker *TNNT2* expression at days 15 without purification. **f**, **g** Immunostaining for the protein expression of *MLC2v* and *MLC2a* in WT and KOs. Scale bar = 25 μm. Data are expressed as means ± S.E.M. of 3 independent experiments

Since the hERG channel protein is primarily expressed on CMs, we used small molecules with clear chemical compositions to induce the differentiation of stem cells into CMs (Fig. 2a); 60-day-old cells were used for western blot, which confirmed the depletion of the hERG protein (Fig. 2b). To further probe the relevance of hERG in heart development, 30-day-old CMs were stained for troponin T (*TNNT2*) and α-actinin (Fig. 2c). Next, flow cytometry showed that both WT and *KCNH2*^*−/−*^ CMs (KO) were nearly 85% *TNNT2-*positive (Fig. 2d, e). Additionally, we performed double immunostaining for *MYL2* and *MYL7* and revealed no significant changes in CM subsets (Fig. 2f, g). These results were consistent with those of a previous study [31]. Taken together, we demonstrated that hERG deficiency did not impact on myocardial differentiation.

### Responses to hERG blockers

**Fig. 3:**
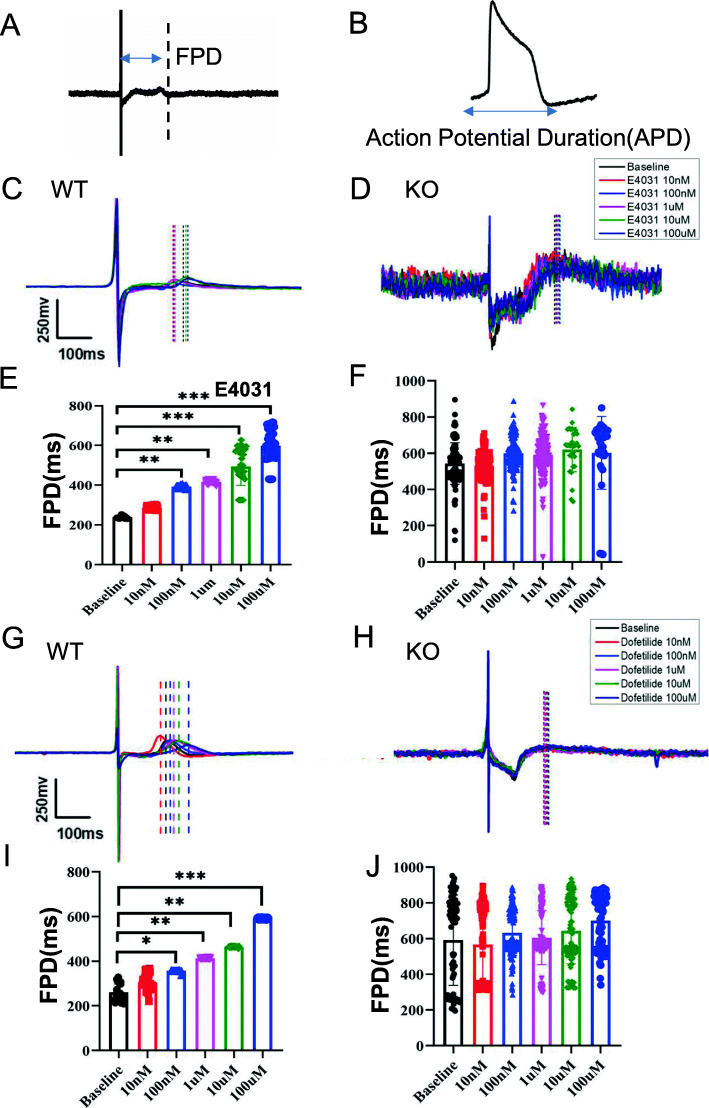
*KCNH2* deletion led to the loss of hERG function. **a**, **b** Schematic diagrams of FPD and APD by MEA processing. **c**, **d** The signals of FPD on different concentrations of E-4031 recorded in WT and KOs. **e**, **f** Quantification of FPD. n = 3 independent experiments, unpaired *t* test. **g**, **h** Signals of FPD on different concentrations of dofetilide recorded in WT and KOs. **i**, **j** Quantification of FPD. *n* = 3 independent experiments, unpaired *t* test. *P* < 0.05 was considered statistically significant (*P < 0.05, **P < 0.01, ***P < 0.001, ****P < 0.0001)

To determine the functions of an hERG-deficient model, the effect of two selective ion channel blockers on spontaneous field potential duration (FPD) was tested. E-4031 (100 nM) [31, 32] treatment caused prolongation in FPD in control CMs (*n* = 24), which demonstrated the presence of functional hERG channels. Moreover, treatment of WT cells with increasing concentrations of E-4031 induced a dose-dependent increase in FPD (Fig. 3c, e). In contrast, the KO (*n* = 24) exhibited insensitivity to different concentrations of E-4031 (Fig. 3d, f). Similar results were obtained with another hERG channel blocker, Dofetilide [33]. The KO exhibited almost no reaction, even with an extended reaction time (Fig. 3g–j). This was expected as E-4031 and Dofetilide act primarily on the IKr current, and this current is absent in hERG-deficient CMs. These results demonstrated that we successfully generated an hERG dysfunction model.

### Irregular rhythm and EAD occurrence

**Fig. 4:**
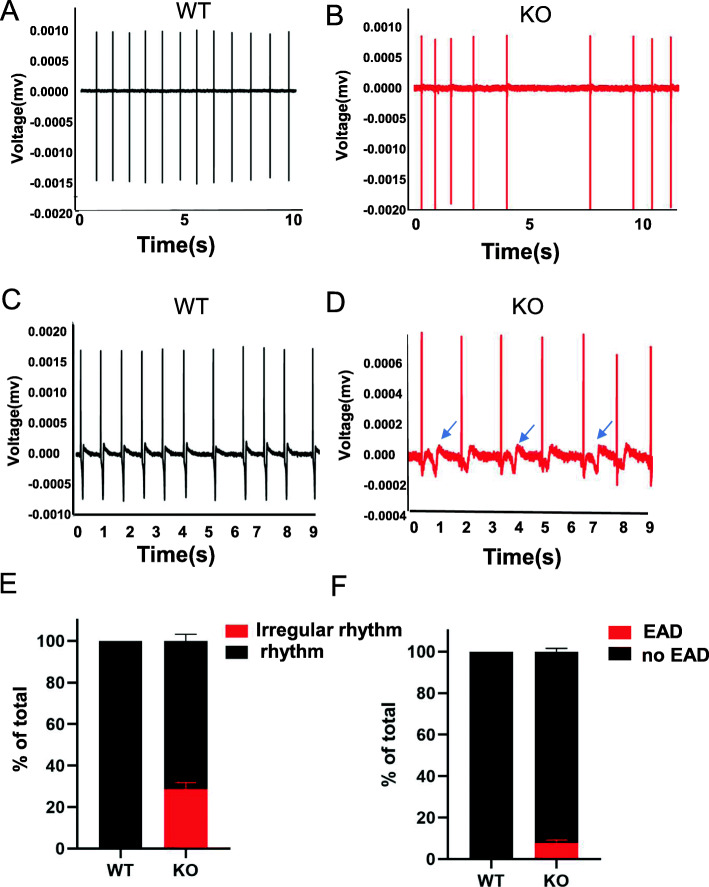
hERG deficiency led to irregular rhythm and EAD. **a**, **b** Representative traces of irregular rhythm were recorded in hERG deficiency. **c**, **d** Representative traces of EAD in FPD. The abnormal signals are labelled with blue arrows. **e**, **f** Quantification of **b** and **d**

Previous studies showed that *KCNH2* loss-of-function mutations cause LQT2, whereas irregular rhythm and EADs are precursors of ventricular arrhythmias in LQTS [34]. We detected differences in hERG-deficient lines at the multicellular level by high-throughput MEA analysis [35] (Fig. S1C). The results implied that hERG-deficient lines (*n* = 5/24) are more prone to irregular rhythm (Fig. 4b, e). Importantly, hERG-deficient lines displayed significant EADs (2/24) (Fig. 4d, f). EAD is a spontaneous membrane depolarisation, and when membrane potential depolarisation reaches a threshold, EAD may trigger action potentials prematurely and cause arrhythmia [19, 36]. Conversely, control CMs (*n* = 24) exhibited no EADs or any other arrhythmogenic activities (Fig. 4a, c). To assess the sensitivity of the hERG-deficient lines to neurohormonal regulation [37], we administered the adrenergic beta-agonist isoproterenol (ISO). The results revealed that ISO exhibited positive chronotropic action. We observed increased beating frequency (Fig. S2A, B), enhanced spike amplitude (Fig. S2D) and shortened FPD (Fig. S2C) with increasing drug concentrations. Overall, in vitro, the hERG-deficient model reproduced important electrophysiological changes that cause ventricular arrhythmia.

### Baseline MEA electrophysiology

**Fig. 5:**
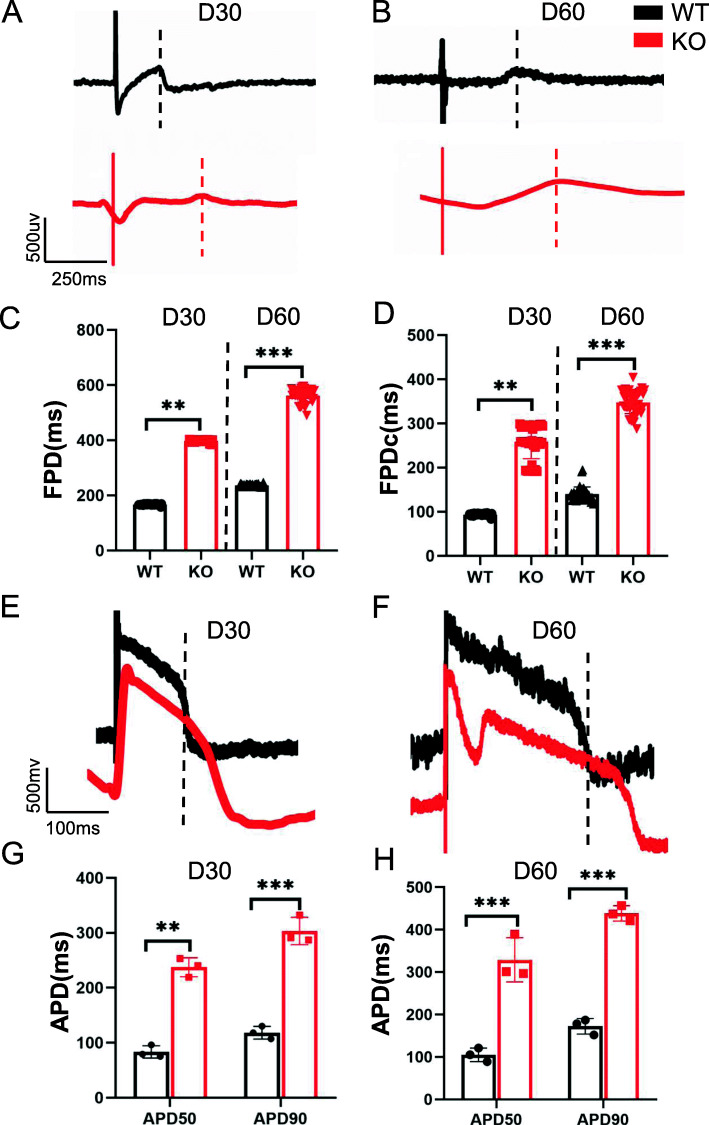
FPD and APD prolongation in hERG-deficient lines. **a**, **b** Signals of FPD recorded on days 30 and 60 by MEA in WT and KOs. **c**, **d** Quantification of FPD and FPDc. *n* = 3 independent experiments, unpaired *t* test. **e**, **f** Recording trace of APD on days 30 and 60 in WT and KOs. **g** Quantification of AP at APD50, APD70 and APD90. *n* = 3 independent experiments, unpaired *t* test. *P* < 0.05 was considered to be statistically significant (*P < 0.05, **P < 0.01, ***P < 0.001, ****P < 0.0001)

To evaluate the baseline electrophysiological measurements of the hERG-deficient model, we selected preliminary mature CMs on day 30 and mature CMs on day 60 for separate testing [38]. Based on the recorded extracellular electrograms and FPD data analysis, we concluded that the FPD in hERG-deficient lines (*n* = 24) was longer than that in the control (*n* = 24) in hESC-CMs (Fig. 5a–c). To identify the baseline value of the hERG-deficient lines more precisely, we used the cell-beating frequency to normalise the FPD to obtain the corrected FPD (FPDc), which was analogous to the corrective QT interval in the ECG (Fig. 5d). Likewise, the results showed marked APD prolongation in hERG-deficient hESC-CMs compared with controls (Fig. 5e–g). Moreover, the same hERG-deficient CMs derived from hPSC showed characteristically prolonged FPD and APD properties (data not shown). The above experimental results demonstrated that the hERG-deficient model exhibited obvious QT prolongation electrophysiological characteristics.

### Responses to other ion channel blockers

**Fig. 6:**
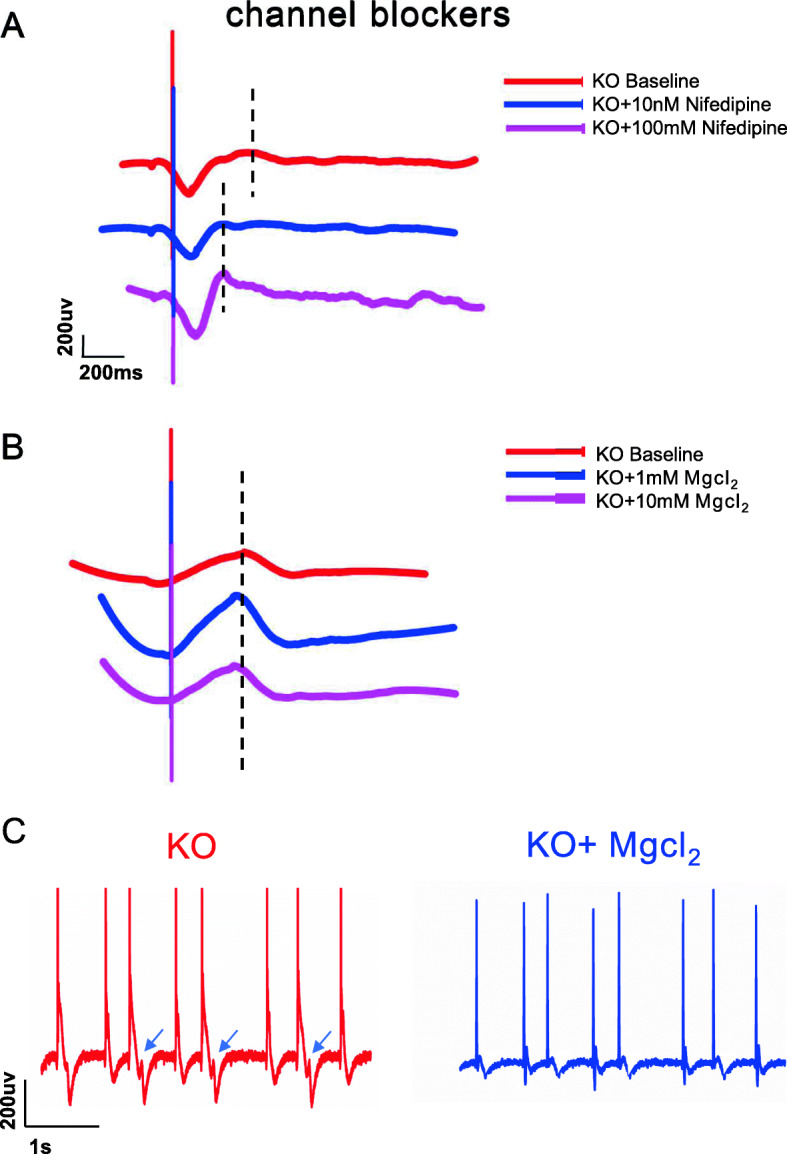
hERG deficiency in response to other ion-channel blockers. **a** Representative FPD of KOs after treatment with nifedipine (*n* = 5). **b** Representative FPD of KOs after treatment with Mgcl_2_ (*n* = 5). **c**, **d** Abrogation of EAD by MgCl_2_ intervention (*n* = 3)

To further characterise the pharmacology of the hERG-deficient model, we assessed cell sensitivity to other ion channel blockers. We first tested nifedipine, a potent dihydropyridine L-type calcium channel blocker [39]. Nifedipine resulted in a substantial reduction in FPD with 10 nM or 100 nM dosages (Fig. 6a). We then tested MgCl_2_ for the clinical treatment of LQTS [40], and the results indicated that MgCl_2_ failed to shorten the QT interval but reduced the EAD development (Fig. 6b). Overall, the hERG-deficient model can be used to screen other ion channel drugs to improve the abnormal phenotype.

## Discussion

In this study, we generated a complete hERG -deficient model to characterise the pathogenesis of LQT2 and its important phenotypes in vitro. Crucially, the hERG-deficient model reproduced pronounced QT prolongation and rhythm disorders. Moreover, our results indicated that other ion channel blockers can partially correct abnormal phenotype. Our model provides an important platform for understanding the fundamental pathogenesis of hERG dysfunction, for defining genotype–phenotype correspondence and promoting drug development.

Three reports of human homozygous hERG mutations have been published, and all cases were related to severe LQTS and even embryonic lethality in infants [41–43]. Some homozygous carriers died in utero, while the ones that survived had severe cardiac electrophysiological disorders. This is of high interest because the same mutation led to different results. One possible hypothesis suggested by these cases is that in vivo even homozygous mutations with serious phenotypes are influenced by additional unknown factors. However, to date, the pathological mechanism of the complete deficiency of hERG is still unclear, and the appropriate methods or models for the study are unavailable. In cases of homozygous hERG mutations, the loss of functional IKr corresponds to ‘human hERG knockout’. Thus, we established an in vitro hERG-hESC-derived CM model to explore the pathological mechanism of the hERG deficiency. One advantage of our model over others was that CMs derived from hESCs and hPSCs reproduced cardiac repolarisation that was strictly controlled by various internal and external ionic currents [44], whereas non-cardiac cell models cannot fully simulate these subtle changes [45]. hESC and hPSC provide a valuable experimental platform for studying ion channel diseases [46]. Although hESC and hPSC are essentially different, such as they are derived from disparate sources, there is considerable consensus regarding the functional similarity of hESC and hPSC [47, 48]. Our data demonstrated that we were able to successfully establish an hERG-deficient hESC-derived CM model using CRISPR/Cas9. Further functional studies were also performed. E-4031 and Dofetilide are the most common selective IKr inhibitors [37]. Expectedly, cell lines with hERG deficiency were less sensitive to E-4031 than control cells. Dofetilide sensitivity was similar in the hERG-deficient model. The establishment of this hERG-deficient model was further supported by drug intervention. Importantly, this in vitro hERG-deficient model may facilitate the understanding of severe LQTS in foetuses.

*KCNH2* is expressed abundantly in CMs where it participates in electrophysiological activities [2]; however, hERG is not believed to be involved in cardiac development [41]. In our study, the CM differentiation capacities of hERG-deficient lines were similar to those of the control. Additionally, no differences in myocardial subtype or myocardial structure were observed. These results agree with previous studies that reported that in humans with hERG homozygous mutations, the heart was structurally normal on echocardiography, with moderate ventricular function. Experiments of catecholamine-induced stress showed that an hERG-deficient cell line had a normal neurohormonal response. Most currents experience developmental maturation in cardiomyocytes. Generally, the QTc interval increases with age [49], and hESC-derived CMs mature over time during in vitro differentiation as they move towards an adult phenotype [50]. Consequently, our experiments included early mature (day 30) and late mature (day 60) CMs. As the CMs matured, the FPD, FPDc and APD on day 60 were longer than those on day 30 in both control and hERG-deficient cells.

*KCNH2* mutations induce hERG dysfunction, which decreases the IKr current and delayed repolarisation. QT prolongation is one of the most important phenotypes. The Schwartz scoring system, which is used for clinical LQTS diagnosis, stipulates that a 460–470 ms QT interval represents medium risk, whereas 480 ms or more indicates a high risk [51]. Our results demonstrate that compared with the control, the FPD, FPDc and APD of the hERG-deficient model were significantly longer. However, these values in hERG-deficient cells were shorter than those reported in other LQT2 hPSC models and clinical values. One reason for this difference may be the inclusion of different cell lines. The cells were derived from different sources, and electrophysiological variability may have been increased by their distinct genetic backgrounds as well as the involvement of other genes in the regulation of electrophysiological activity [31]. Different studies in the past reported the same point mutation A164V in the pore region, and the difference between APD70 and APD90 of the A164V hPSC-CM from different patients was more than 200 ms [19, 20]. These results also reflected that the functional changes in point mutations are unstable and difficult to standardise. Moreover, we used MEA analysis to detect electrophysiological activity at the multicellular level [26]. Compared with patch-clamp, the contact between cells in syncytium may result in other ion repolarisation compensation mechanisms to protect the abnormal milieu from serious deviation. Another important consideration is the beat times of hESC-derived CMs, which generally range from 30 to 40 in vitro [19]. This is lower than the normal adult heart rate. Therefore, FPDc is much shorter than FPD.

Since our study compared different hERG statuses in the same system, an unambiguous genotype–phenotype relationship was ascertained. Many pathological mechanisms of heterozygous point mutations have been investigated [4], but the role of WT alleles in cases of heterozygous mutations is still unclear. Furthermore, their studies have not yet been stated clearly the mechanism of complete hERG deletion. hERG1b, a subunit that interacts with hERG (hERG1a), accounts for 19% of the total hERG gene expression in the right atrium and 12% in the left ventricle. Due to the unique ‘RXR’ endoplasmic reticulum retention signal at the hERG1b N-terminus, it cannot be expressed on the membrane alone [52]. Using the masking effect of hERG1a, an hERG1a/hERG1b composite channel is formed to complete the normal membrane deposition and effectively produce a stronger repolarisation current than the hERG1a complex alone. For this reason, the normal WT allele in cases with heterozygous mutations may also contribute to repolarisation. Thus, homozygous hERG deficiency is a more explicit and representative genotype–phenotype model for research. The overriding point in this aspect is that the hERG knockout model was easy to obtain and that the functional changes in hERG-deficient cell lines were more stable than that of point mutations. In addition, it also covered the functional changes in all mutations, which is a suitable model for studying QT prolongation and irregular rhythms. Furthermore, we captured irregular rhythms in this hERG-deficient model. Concomitantly, EAD also appeared. This is roughly analogous to the 2:1 atrioventricular block that occurs in homozygous hERG patients. Using the deficiency model, we also conducted pharmacological studies. We selected two drugs to treat homozygous hERG probands: Nifedipine and MgCl_2_. Our results showed that both 10-nM and 100-nM Nifedipine substantially shortened the QT interval. While MgCl_2_ failed to change the QT interval, it reduced the appearance of EADs. This is consistent with previous reports that stated that MgCl_2_ suppressed depolarisation-induced automaticity when APD was fixed [53].

## Conclusions

In summary, we generated an hERG-deficient in vitro model using CRISPR/Cas9. The model exhibited marked QT prolongation, arrhythmia and sensitivity to other ion channel blockers and serves as an important tool to increase our understanding of the fundamental pathological mechanism of hERG dysfunction, define genotype–phenotype correspondence and facilitate drug development.

## Supplementary Information

**Additional file 1: Figure S1.** hERG deficiency did not affect pluripotency of hESCs. a: Brightfield images showing the morphology of WT and *KCNH2*^*−/−*^; b: *In vitro* differentiation of *KCNH2*^*−/−*^ of ectoderm, endoderm, and mesoderm; **c**: Brightfield images showing CMs attachment to an electrode piece of MEA. **Figure S2.** Neurohormonal responses. a, b: After isoproterenol intervention, representative FPD recording in KOs; c: Signals of FPD on different concentrations of isoproterenol in KOs; d: Quantification of spike amplitude with different concentrations of isoproterenol. n = 3 independent experiments, *t*-test. *P* < 0.05 was considered to be statistically significant (*P < 0.05, **P < 0.01, ***P < 0.001, ****P < 0.0001).

## Data Availability

All data generated or analysed during this study are included in this published article.

## Abbreviations

LQT2: Long-QT syndrome type 2
hERG: *Human ether-a-go-go-related gene*
CM: Cardiomyocyte
hESC: Human embryonic stem cell
MEA: Microelectrode array
IKr: Delayed rectifier K^+^ channel
LQTS: Long-QT syndrome
APD: Action potential duration
hPSCs: Human pluripotent stem cells
WT: Wild-type
EAD: Early afterdepolarisation
MgCl_2_: Magnesium chloride
sgRNA: Single-stranded guide RNA
PCR: Polymerase chain reaction
PFA: Paraformaldehyde
*TNNT2*: Troponin T
KO: *KCNH2*^*−/−*^ CMs
FPD: Field potential duration
ISO: Isoproterenol
FPDc: Corrected FPD
