# Supplementary figures and images for: hERG-deficient human embryonic stem cell-derived cardiomyocytes for modelling QT prolongation
Source: Stem Cell Res Ther. 2021 May 7;12:278. doi: 10.1186/s13287-021-02346-1 (PMC8103639; doi:10.1186/s13287-021-02346-1)

Fig.S1 hERG deficiency did not affect pluripotency of hESCs

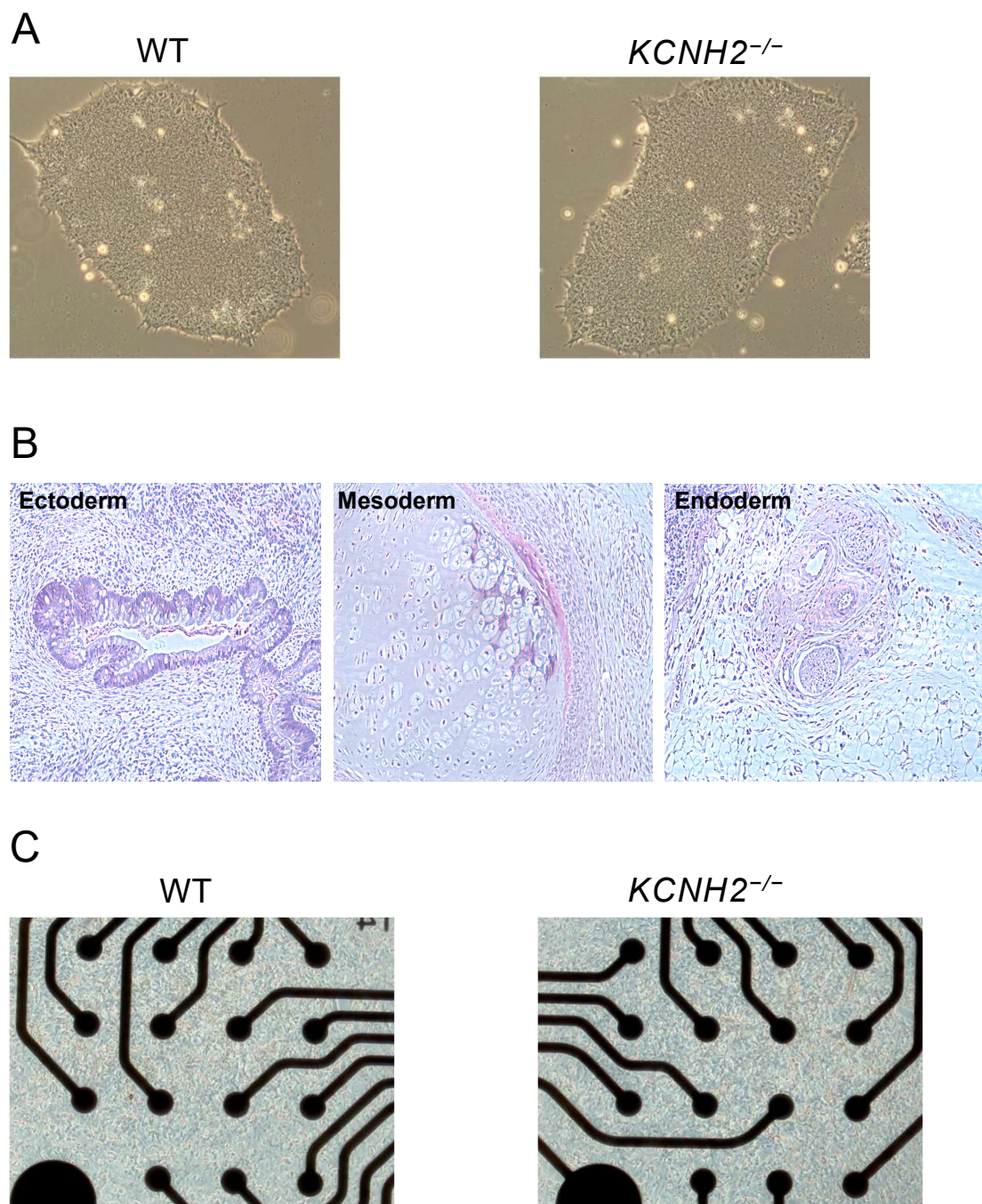

Fig.S2 Neurohormonal responses

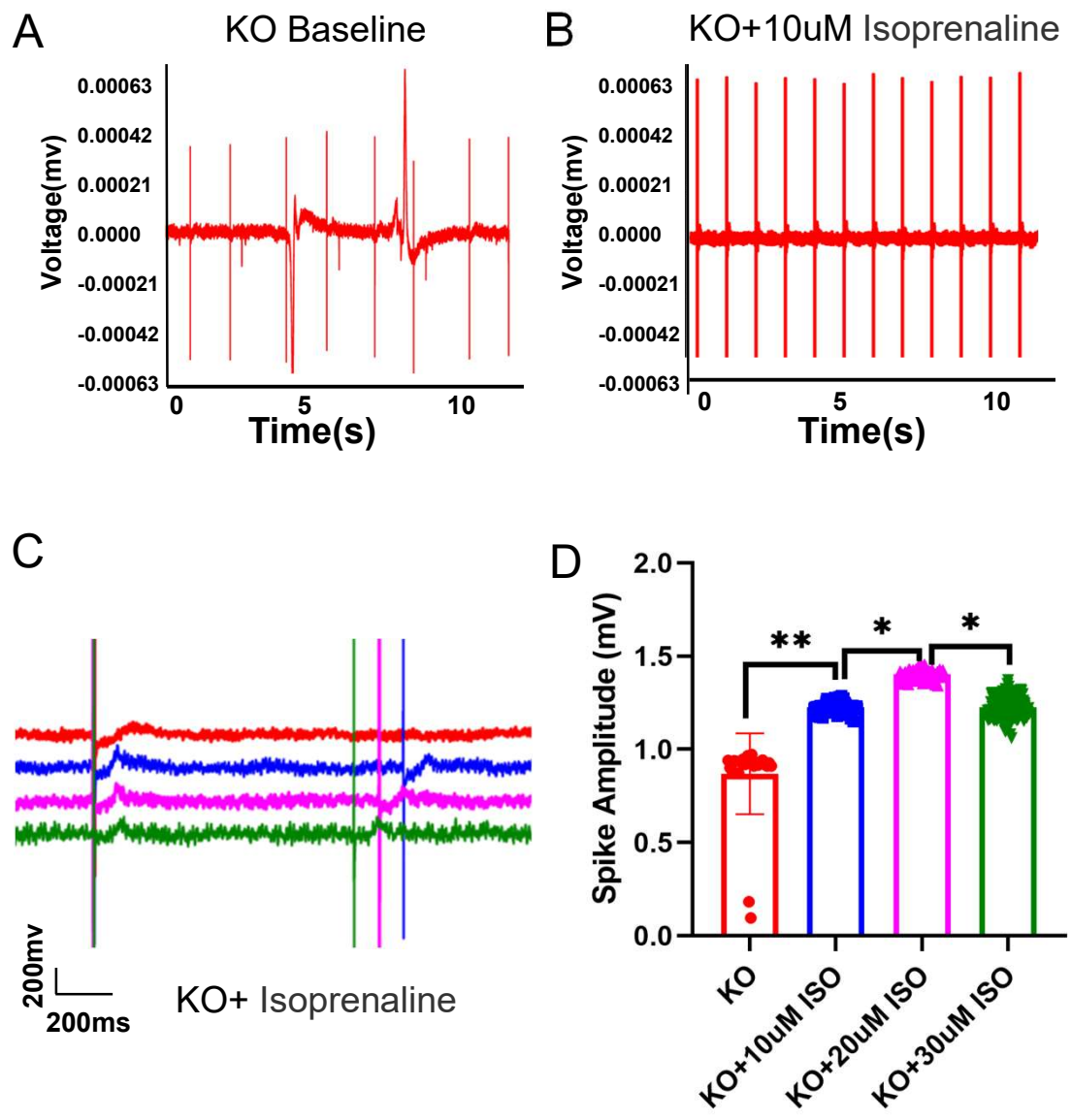

Supplement: Supplementary file 1 — Additional file 1: Figure S1. hERG deficiency did not affect pluripotency of hESCs. a: Brightfield images showing the morphology of WT and KCNH2−/−; b: In vitro differentiation of KCNH2−/− of ectoderm, endoderm, and mesoderm; c: Brightfield images showing CMs attachment to an electrode piece of MEA. Figure S2. Neurohormonal responses. a, b: After isoproterenol intervention, representative FPD recording in KOs; c: Signals of FPD on different concentrations of isoproterenol in KOs; d: Quantification of spike amplitude with different concentrations of isoproterenol. n = 3 independent experiments, t-test. P < 0.05 was considered to be statistically significant (*P < 0.05, **P < 0.01, ***P < 0.001, ****P < 0.0001). [file 13287_2021_2346_MOESM1_ESM.pdf]
